# Structural basis for the transformation pathways of the sodium naproxen anhydrate–hydrate system

**DOI:** 10.1107/S2052252514015450

**Published:** 2014-08-20

**Authors:** Andrew D. Bond, Claus Cornett, Flemming H. Larsen, Haiyan Qu, Dhara Raijada, Jukka Rantanen

**Affiliations:** a Department of Pharmacy, University of Copenhagen, Universitetsparken 2, Copenhagen DK-2100, Denmark; b Department of Food Science, University of Copenhagen, Rolighedsvej 30, Frederiksberg DK-1958, Denmark; c Department of Chemical Engineering, Biotechnology and Environmental Technology, University of Southern Denmark, Niels Bohrs Alle 1, Odense DK-5230, Denmark

**Keywords:** pharmaceutical, hydrate, X-ray diffraction, solid-state NMR, DFT-D

## Abstract

Crystal structures are presented for two dihydrate polymorphs (DH-I and DH-II) of the non-steroidal anti-inflammatory drug sodium (*S*)-naproxen. The structure of DH-I is determined from twinned single crystals obtained by solution crystallization. DH-II is obtained by solid-state routes, and its structure is derived using powder X-ray diffraction, solid-state ^13^C and ^23^Na MAS NMR, and molecular modelling. The validity of both structures is supported by dispersion-corrected density functional theory (DFT-D) calculations. The structures of DH-I and DH-II, and in particular their relationships to the monohydrate (MH) and anhydrate (AH) structures, provide a basis to rationalize the observed transformation pathways in the sodium (*S*)-naproxen anhydrate–hydrate system. All structures contain Na^+^/carboxylate/H_2_O sections, alternating with sections containing the naproxen molecules. The structure of DH-I is essentially identical to MH in the naproxen region, containing face-to-face arrangements of the naphthalene rings, whereas the structure of DH-II is comparable to AH in the naproxen region, containing edge-to-face arrangements of the naphthalene rings. This structural similarity permits topotactic transformation between AH and DH-II, and between MH and DH-I, but requires re-organization of the naproxen molecules for transformation between any other pair of structures. The topotactic pathways dominate at room temperature or below, while the non-topotactic pathways become active at higher temperatures. Thermochemical data for the dehydration processes are rationalized in the light of this new structural information.

## Abstract 2

Relationships between the crystal structures of two polymorphs of sodium naproxen dihydrate and its monohydrate and anhydrate phases provide a basis to rationalize the observed transformation pathways in the sodium (*S*)-naproxen anhydrate–hydrate system.

## 1. Introduction   

The correlation of molecular-level structure with observed physicochemical properties is a fundamental activity in the chemical sciences. Our interest lies principally with pharmaceutical compounds, for which robust physicochemical understanding is paramount (Connelly *et al.*, 2011 ▶). According to regulatory guidelines (ICH, 2000 ▶; US-FDA, 2007 ▶), pharmaceutical companies are encouraged to search for alternative solid forms of active pharmaceutical ingredients (APIs), and to assess the risks associated with solid-state factors such as potential polymorphic transformations. In this context, hydrates have a particular importance because of the ubiquitous nature of water in our environment. Investigation and understanding of hydrates with pharmaceutical relevance, and especially the transformations that occur between different hydration states in anhydrate–hydrate systems, is therefore of significant interest within pharmaceutical materials science (Griesser, 2006 ▶; Zhang *et al.*, 2004 ▶; Reutzel-Edens *et al.*, 2003 ▶; Roy *et al.*, 2008 ▶).

In this paper we consider the non-steroidal anti-inflammatory drug (NSAID) sodium (*S*)-naproxen (chemical structure shown in Scheme 1 with applied labelling scheme). The compound is known to exist as an anhydrate (AH), a monohydrate (MH), two dihydrate polymorphs (DH-I and DH-II) and a tetrahydrate (TH) (Di Martino *et al.*, 2001 ▶, 2007 ▶; Kim & Rousseau, 2004 ▶; Malaj *et al.*, 2009 ▶; Raijada *et al.*, 2013 ▶). A thorough empirical study of the thermodynamic and kinetic aspects of dehydration in the system has been reported by Malaj *et al.* (2009 ▶). Those authors refer to the various phases as ASN (= AH), MSN (= MH), CSN (= DH-I), DSN (= DH-II) and TSN (= TH). We have also previously studied the transformation pathways for the system using multi-temperature dynamic vapour sorption (DVS) and variable-temperature/humidity powder X-ray diffraction (PXRD), and we have identified routes to isolate bulk samples of the various phases (Raijada *et al.*, 2013 ▶).
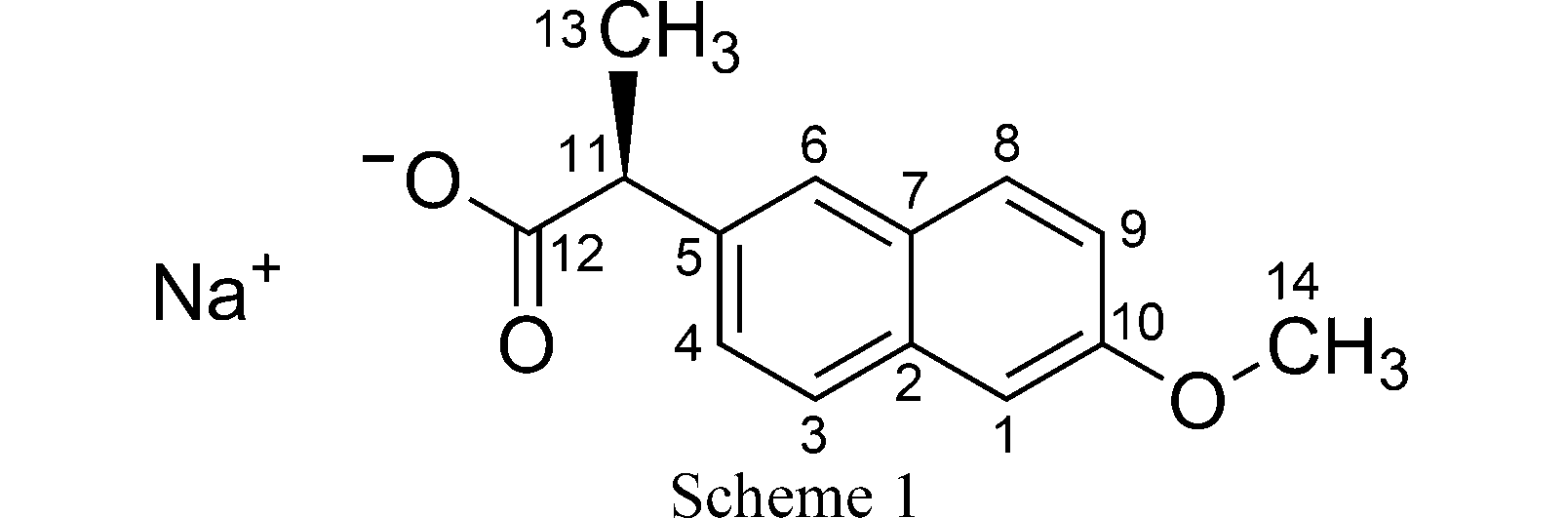


**Figure 1:**
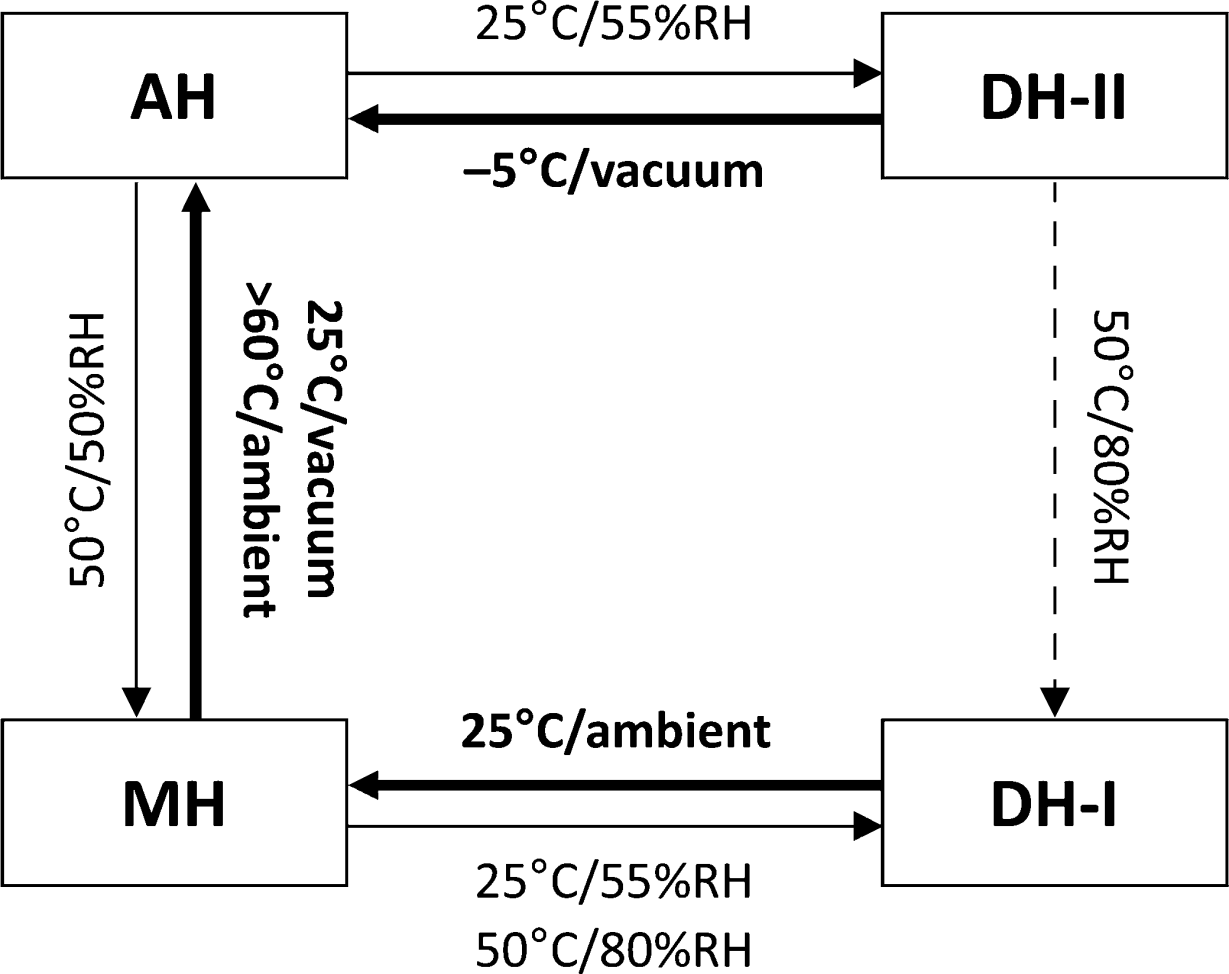
Summary of the transformation pathways for the sodium naproxen anhydrate–hydrate system. Hydration is shown with normal text/arrows, dehydration is shown with bold text/arrows.

A summary of the transformation behaviour is shown in Fig. 1 ▶. For hydration of AH, different pathways are followed depending on the temperature. At 25°C/55% relative humidity AH transforms to DH-II, while at 50°C/50% relative humidity AH transforms to MH. Hydration of MH proceeds to DH-I, either at 25°C/55% relative humidity or 50°C/80% relative humidity. These results are obtained from variable-temperature/humidity PXRD experiments. Corresponding behaviour is observed on dehydration of the dihydrate phases. Malaj *et al.* (2009 ▶) have reported that DH-I transforms sequentially to MH then to AH, while DH-II transforms immediately to AH under isothermal dehydration conditions in the temperature range 22–37°C. We concur with these results and have also found similar behaviour for dehydration at lower temperature (−5°C) under vacuum (Raijada *et al.*, 2013 ▶). Thermogravimetric analysis (TGA) shows that both DH-I and DH-II exhibit a plateau corresponding to MH before proceeding to AH, but MH persists over a larger temperature range for DH-I compared with DH-II (Malaj *et al.*, 2009 ▶; Raijada *et al.*, 2013 ▶).

Our aim in this paper is to link the observed hydration/dehydration behaviour to the molecular-level solid-state structures. To date, crystal structures have been published for AH (Kim *et al.*, 2004 ▶) and MH (Kim *et al.*, 1990 ▶). A structure described as a heminonahydrate, NS·4.5H_2_O, has also been reported (Burgess *et al.*, 2012 ▶), which is disordered and which we have re-interpreted as TH (Bond *et al.*, 2013 ▶). Structural information for the polymorphic dihydrate, however, has so far not been reported. We discuss the structures of the two DH polymorphs in this paper, and show that they provide a clear basis to understand the transformation behaviour of the system. The structures presented here complete the (currently known) structural landscape of the sodium naproxen anhydrate–hydrate system, and illustrate the value of a complete structural picture for robust physicochemical understanding.

## 2. Experimental   

### 2.1. Materials   

Sodium (*S*)-naproxen anhydrate (AH; USP grade) was obtained from Divi’s Laboratories Ltd, India. Methods for preparation of the various bulk phases have been described previously (Raijada *et al.*, 2013 ▶). Single crystals of DH-I were obtained by dissolving 500 mg of AH in 79 mol % EtOH (4.62 ml EtOH + 0.38 ml H_2_O) at 60°C. The solution was filtered to remove any undissolved residues of AH then allowed to cool to room temperature. The first single crystals to appear were transferred rapidly from the mother liquor to perfluoropolyether oil, then into the N_2_ cryostream (at 150 K) on the diffractometer. Several crystals from different batches were examined, with comparable results.

### 2.2. X-ray diffraction   

Single-crystal X-ray diffraction data were collected using a Bruker–Nonius X8-APEXII instrument equipped with graphite-monochromated Mo *K*α radiation (λ = 0.7107 Å). Data were collected at 150 K under an N_2_ cryostream in an effort to minimize dehydration during the data collection. Structure solution and refinement were carried out using *SHELXL* (Sheldrick, 2008 ▶). Powder X-ray diffraction data were collected using a Panalytical X’Pert Pro instrument equipped with non-monochromated Cu *K*α radiation (average λ = 1.5418 Å). Data were collected in either flat-plate reflection or transmission capillary modes. Preliminary data analysis was carried out using *HighScorePlus* (Panalytical, 2012 ▶), and pattern indexing was achieved using *DICVOL* (Boultif & Louër, 2004 ▶). Rietveld refinements were performed with *TOPAS Academic*, Version 4.1 (Coelho, 2007 ▶) using the *DASH* (David *et al.*, 2006 ▶) interface for construction of the initial input files.

### 2.3. Solid-state ^23^Na and ^13^C MAS NMR   

Solid-state MAS NMR spectra were recorded on a Bruker Avance 400 spectrometer operating at Larmor frequencies of 100.62, 105.85 and 400.13 MHz for ^13^C, ^23^Na and ^1^H, respectively, using a double-tuned CP/MAS probe. A ^23^Na MAS NMR spectrum was also recorded for AH on a Bruker Avance-II 700 spectrometer operating at 185.15 MHz. Samples were loaded in sealed tubes to avoid dehydration. The measurement temperature was 313 K. Detailed experimental conditions are provided in the supporting information. All data were initially processed using *TOPSPIN* 2.1 (Bruker, 2008 ▶) then transferred to *MATLAB* (Mathworks, 2000 ▶) to set up figures. Numerical simulations and iterative fitting of the experimental ^23^Na MAS spectra to extract isotropic chemical shifts and quadrupolar parameters for ^23^Na were performed using a modified version of the software described in Larsen *et al.* (1998 ▶), assuming ideal RF-excitation. Additional experimental methods are discussed in Bennett (1995 ▶), Brown (1997 ▶), Delaglio (1995 ▶) and Peersen (1993 ▶).

### 2.4. Computational methods   

Energy minimization of the crystal structures was carried out by dispersion-corrected density functional theory (DFT-D) calculations, using the *CASTEP* module (Clark *et al.*, 2005 ▶) within *Materials Studio* (Accelrys, 2011 ▶). The PBE functional was applied (Perdew *et al.*, 1996 ▶) with a plane-wave cut-off energy of 520 eV and a dispersion correction according to Grimme (2006 ▶). All other parameters were set to the ‘fine’ defaults within *Materials Studio*. All atomic coordinates and unit-cell parameters were allowed to optimize within the constraints of the crystal system. A validation study has established that this methodology can be expected to reproduce the geometry of correct molecular crystal structures with an average r.m.s. deviation of *ca* 0.08 Å for the non-H atoms (van de Streek & Neumann, 2010 ▶).

## 3. Results and discussion   

### 3.1. Crystal structures of AH and MH   

**Figure 2:**
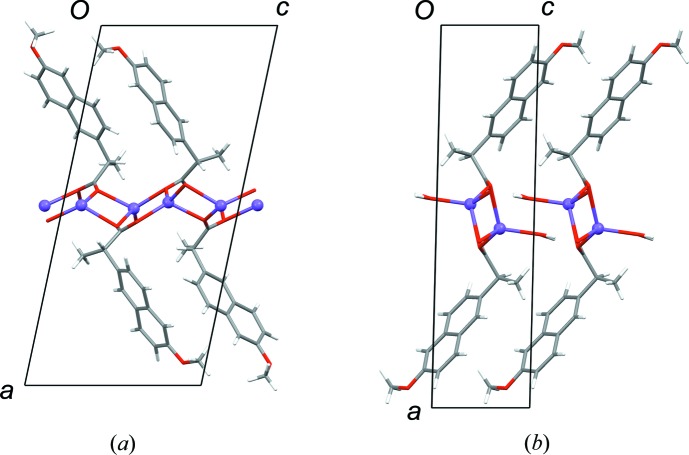
View of the structures of (*a*) AH and (*b*) MH along the *b* axis. Data are taken from Kim *et al.* (1990 ▶, 2004 ▶).

**Figure 3:**
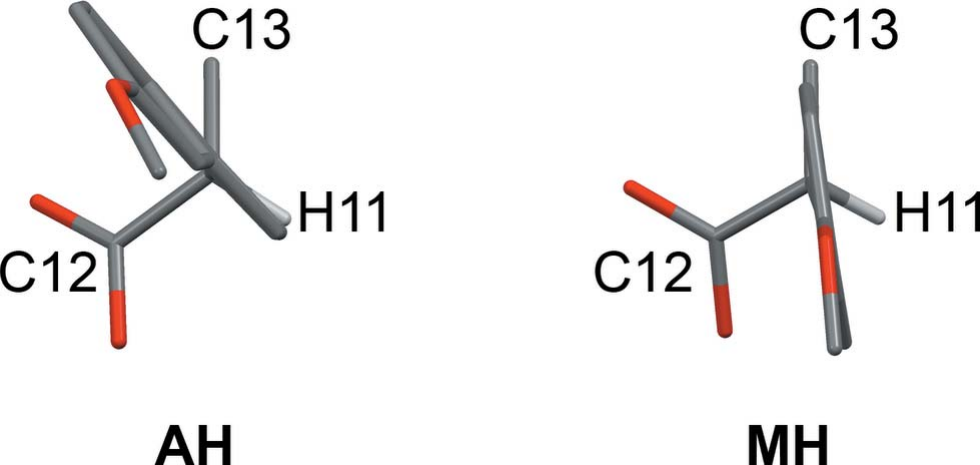
Projection of the naproxen molecule approximately along the C5—C11 bond in AH and MH, showing two different orientations for the naphthalene ring. H atoms (except H11) are omitted.

**Figure 4:**
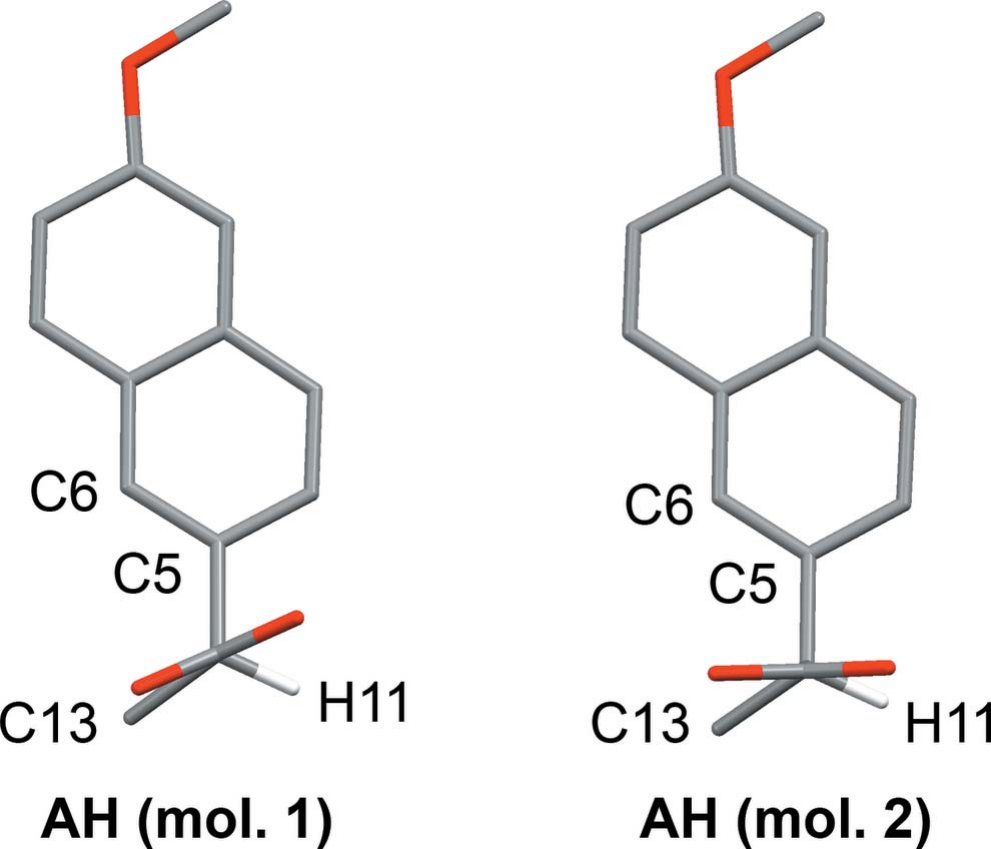
Projection of the two crystallographically independent naproxen molecules in AH approximately along the C11—C12 bond, showing two different orientations for the carboxyl group. H atoms (except H11) are not shown. The conformation in MH is comparable to that of AH (mol. 2).

The structures of AH (Kim *et al.*, 2004 ▶) and MH (Kim *et al.*, 1990 ▶) are layered, containing Na^+^/carboxylate/(H_2_O) sections alternating with sections containing the naproxen molecules (Fig. 2 ▶). For consistent discussion of the structures, we refer to the atomic sites as indicated in Scheme 1, and we transform the published unit-cell setting for AH so that the layers lie parallel to the (100) planes (see supporting information for structures in CIF format). In AH (Fig. 2 ▶) the carboxylate groups adopt two different coordination modes: Na^+^–(μ-O)–Na^+^ and Na^+^–[O–C–O]–Na^+^. The latter define polymeric ribbons along the crystallographic *b* axis. The Na^+^/carboxylate sections are locally centrosymmetric. If the naproxen molecules are deleted so that only the Na^+^ ions and carboxyl groups remain, the structure can be described in space group *P*2_1_/*c* with one crystallographically distinct Na^+^ ion and one carboxylate group. Reduction of the symmetry to *P*2_1_ arises due to the naproxen molecules, which adopt an edge-to-face type arrangement (Fig. 2 ▶). The two crystallographically distinct naproxen molecules display slightly different molecular conformations in the propionate side chain. In both molecules, the naphthalene ring plane lies approximately eclipsed with the C11—H11 bond when viewed in projection along the C5—C11 bond (Fig. 3 ▶). Projection along the C11—C12 bond shows that the carboxyl group in one molecule is eclipsed with the C11—C13 bond, while it lies approximately perpendicular to the C11—C5 bond in the other molecule (Fig. 4 ▶). DFT-D minimization of the published AH crystal structure results in an r.m.s. Cartesian displacement of 0.15 Å for the non-H atoms, which is consistent with expectations for a correct room-temperature structure.

The MH structure (Fig. 2 ▶) contains one-dimensional polymeric ribbons along the crystallographic *b* axis, formed by Na^+^–[O–C–O]–Na^+^ links, which are closely comparable to those in AH (compare the *b* dimensions in Table 1 ▶). The ribbons are paired through square-shaped Na^+^–(μ-O)_2_–Na^+^ units, in which the μ-O linkages are provided by the carboxyl groups. The water molecules project to either side, linking the ribbons along the *a* axis through O—H⋯O hydrogen bonds. The Na^+^/carboxylate/H_2_O sections contain local inversion centres that intersect the crystallographic 2_1_ screw axes so that the space group approximates *P*2_1_/*m*. Again, the inversion symmetry is broken by the naproxen molecules, which in this case adopt a face-to-face type arrangement, with all naphthalene ring planes parallel. The Na^+^ coordination geometry resembles square-based pyramidal, with Na^+^ lying out of the approximate square plane. In contrast to AH, the ring planes of the naproxen molecules lie approximately eclipsed with the C11—C13 bond when viewed in projection along the C10—C11 bond (Fig. 3 ▶). The orientation of the carboxyl group is perpendicular to the C11—C5 bond, as shown for AH (mol. 2) in Fig. 4 ▶. DFT-D minimization of the published MH crystal structure results in an r.m.s. Cartesian displacement of 0.15 Å for the non-H atoms, which is again consistent with expectations for a correct room-temperature structure.

### 3.2. Solid-state ^13^C and ^23^Na NMR   

**Figure 5:**
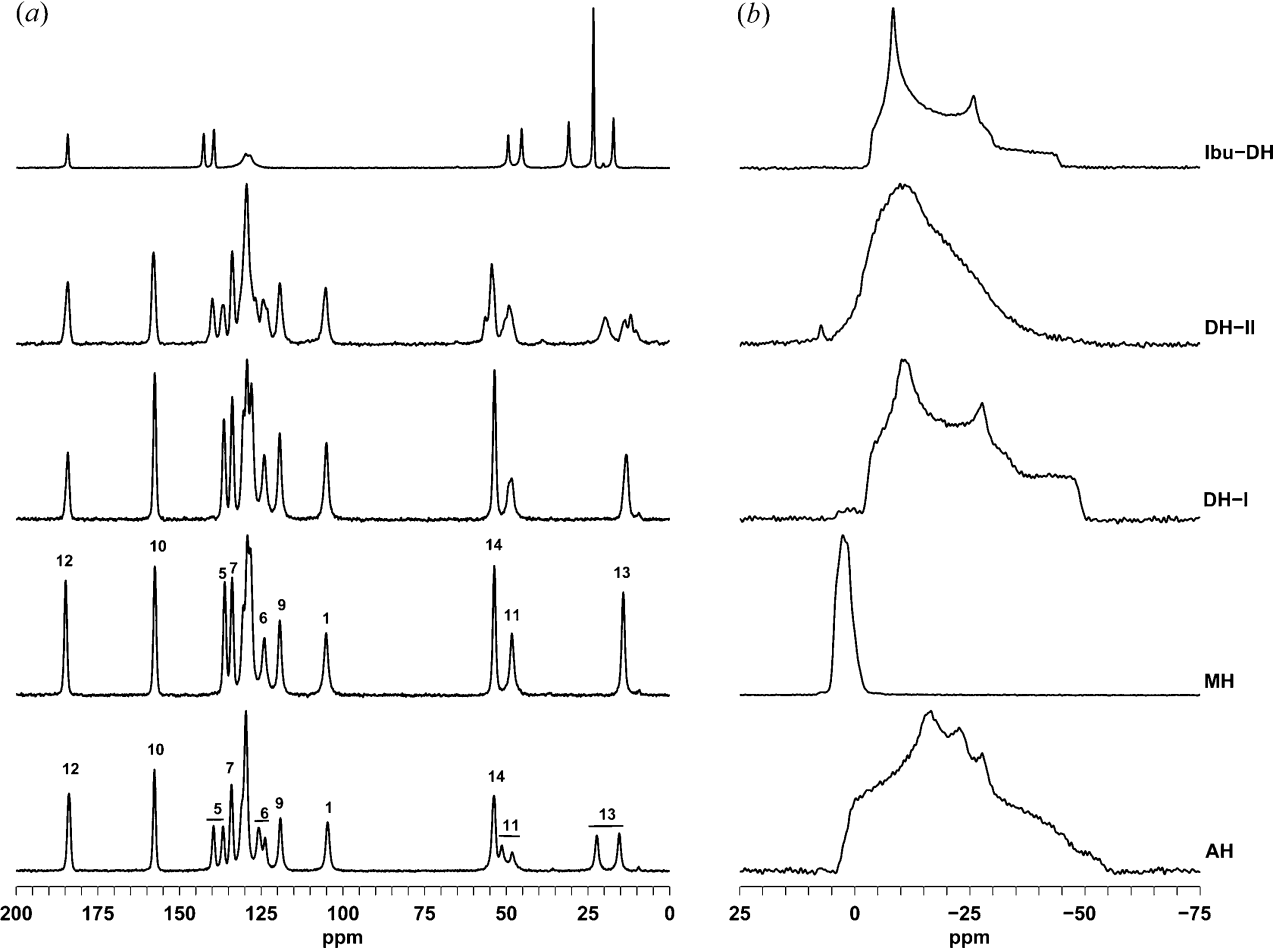
Solid-state ^13^C CP/MAS (column A) and ^23^Na (column B) MAS NMR spectra measured at 313 K. Resonance assignments are shown for AH and MH (labelling according to Scheme 1). The assignments for DH-I and DH-II are clear by analogy. Spectra are also shown for sodium ibuprofen dihydrate (top), which has a similar structure to DH-II in the Na^+^/H_2_O region (see text).

^13^C CP/MAS NMR spectra have been reported previously for AH after exposure to various degrees of humidity (Di Martino *et al.*, 2007 ▶) and ^23^Na MAS NMR have also been reported for AH and MH (Burgess *et al.*, 2012 ▶). Our ^23^Na MAS spectra for all phases (Fig. 5 ▶) demonstrate that the quadrupolar tensor of the ^23^Na site(s) is highly sensitive towards the hydration state. The parameters in Table 2 ▶ (obtained by iterative fitting of the experimental spectra; see supporting information) show that the quadrupolar coupling constant *C*_Q_ is 2.3 MHz or greater for AH and both DH forms, while it is close to 1.0 MHz for MH. The presence of two ^23^Na sites in AH, as indicated by the crystal structure (Kim *et al.*, 2004 ▶), was established by simultaneous iterative fitting of spectra recorded at 9.4 and 16.4 T. A 3Q-MAS spectrum recorded at 9.4 T did not resolve the two sites. The two sites have essentially identical chemical shifts but different quadrupolar parameters, in accordance with previous reports (Burgess *et al.*, 2012 ▶). All of the other phases show apparently only one ^23^Na site. For DH-II an almost featureless ^23^Na MAS lineshape suggests either a disordered structure or a range of mutually exchanging configurations. The contours in the ^23^Na 3Q-MAS spectrum (see supporting information) mainly indicate a distribution in quadrupolar tensor parameters rather than a distribution of chemical shifts.

In the ^13^C CP/MAS NMR spectra (Fig. 5 ▶), AH exhibits two resolved resonances for each of the sites C5, C6, C11 and C13 (labelling as in Scheme 1), whereas only one resonance is observed for these sites in MH. This reveals the difference between the molecular conformations in the region of the propionate side chain (Figs. 3 ▶ and 4 ▶) and the edge-on arrangement of the naproxen molecules in AH compared with the parallel arrangement in MH. The ^13^C spectrum of DH-I is similar to MH, indicating that the parallel arrangement of naproxen molecules is also present in DH-I. However, the resonances for the propionate side chain are noticeably broader for DH-I, which may indicate some degree of structural variation in this region. For DH-II, the ^13^C CP/MAS spectrum resembles that of AH, thereby indicating an edge-on arrangement for the naproxen molecules. Again, the lines are slightly broader for DH-II compared with AH, which may indicate some degree of disorder. It should be noted that the NMR spectra in Fig. 5 ▶ are measured at 313 K, so they may be influenced by dynamic phenomena.

### 3.3. Crystal structure of DH-I   

Numerous solution-grown crystals of DH-I were examined, and all displayed indications of twinning/disorder (discussed further below). The structure was eventually obtained after data integration using a single component in one crystal, although subsequent structure refinement was problematic. In particular, the data:parameter ratio is low (*ca* 4), on account of limited observable data and the low symmetry of the structure, and it was necessary to apply restraints to all bond distances and angles in order to maintain a reasonable geometry. The problems with the single-crystal analysis most likely also reflect some degree of dehydration during the transfer of the crystals from the mother liquor to the N_2_ cryostream and/or in the course of the data collection. The validity of the established structure is supported by DFT-D minimization, which results in an r.m.s. Cartesian displacement of 0.14 Å for the non-H atoms, comparable to that obtained for minimization of AH and MH. Comparison to the PXRD pattern of the bulk sample by Rietveld refinement also provides a satisfactory fit (see supporting information).

**Figure 6:**
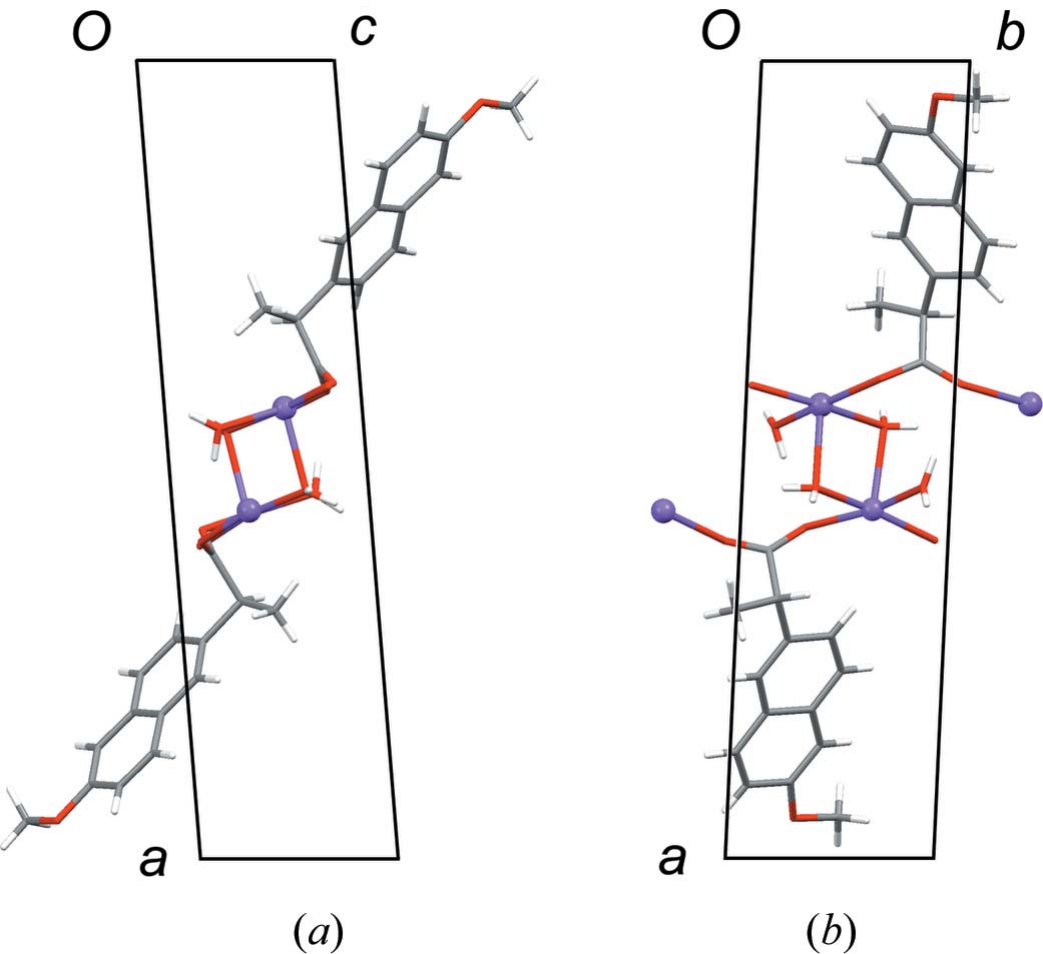
Structure of DH-I. The view along the *b* axis (*a*) is along the direction of propagation of the polymeric ribbon. The view along the *c* axis (*b*) shows one square-shaped Na^+^–(μ-OH_2_)_2_–Na^+^ unit and the square-pyramidal coordination geometry of Na^+^.

The structure of DH-I closely resembles that of MH. In particular, the structures have very similar unit cells (Table 1 ▶) and they are essentially identical in the regions of the naproxen molecules, consistent with the information deduced from the ^13^C CP/MAS NMR spectra. The naproxen regions exhibit local 2_1_ symmetry, and local inversion symmetry also exists within the Na^+^/carboxylate/H_2_O sections. The 2_1_ symmetry is broken by the Na^+^/carboxylate/H_2_O sections and the inversion symmetry is broken by the naproxen molecules, so that the crystallographic symmetry is reduced to *P*1 with two formula units in the asymmetric unit. Similar examples of pseudosymmetry have been noted in the context of isostructural racemic and enantiomeric crystals (Zhang & Grant, 2005 ▶). The molecular conformations of the two crystallographically distinct naproxen molecules are closely comparable, and identical to that in MH. The ring plane of the naproxen molecule lies eclipsed with the C11—C13 bond (Fig. 3 ▶), and the carboxyl group is perpendicular to the C5—C11 bond (Fig. 4 ▶). As for AH and MH, the DH-I structure contains one-dimensional polymeric ribbons along the *b* axis, cross-linked by square-shaped Na^+^–(μ-OH_2_)_2_–Na^+^ units. The linking units are geometrically similar to those in AH, except that the μ-O bridges in DH-I are formed by water molecules (Fig. 6 ▶) rather than carboxyl O atoms. The non-bridging H_2_O molecules form O—H⋯O hydrogen bonds between ribbons. The Na^+^ coordination geometry is close to regular square-based pyramidal, with Na^+^ in the square plane. Despite the formal crystallographic inequivalence of the two Na^+^ sites, the pseudosymmetry means that their local environments are essentially identical, which can account for the observation of a single site in the ^23^Na NMR.

### 3.4. Disorder and twinning in DH-I   

**Figure 7:**
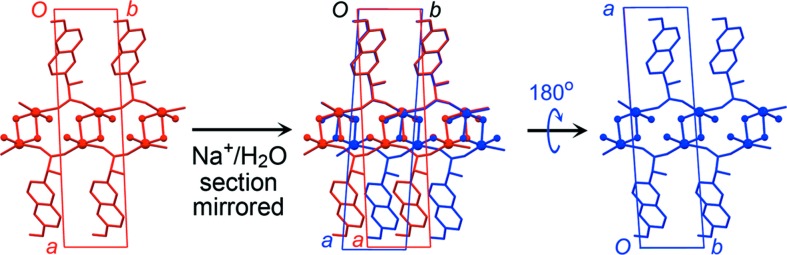
Probable twinning mechanism in DH-I. The Na^+^/H_2_O section is mirrored perpendicular to *b*, with an accompanying shift of ½*b* for the bottom layer of naproxen molecules. The resulting structure (blue) is identical to the starting structure (red) rotated 180° around *b*.

Reconstructed precession images for DH-I (supporting information) appear ordered for 0*kl*, 1*kl* *etc.*, indicative of regular two-dimensional layers in the structure parallel to the (100) planes. The disorder is evident in the diffraction pattern as reflections split along a*. The probable origin of this can be viewed as a result of the pseudosymmetry. Combination of the local 2_1_ operator that relates the naproxen molecules (
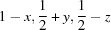
) with the local inversion operator (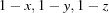) within the Na^+^/H_2_O sections generates a mirror operator (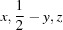). This local mirror can be applied to the Na^+^/H_2_O section to reverse the orientation of the square-shaped Na^+^–(μ-OH_2_)_2_–Na^+^ units relative to the *b* axis (Figs. 7 ▶ and 8 ▶). If this occurs, the next layer of naproxen molecules is shifted by ½*b* compared with its expected position in order to maintain identical chemical contacts (Fig. 7 ▶). A ‘fault’ of this kind corresponds to twinning of the structure by 180° rotation around the *b* axis. DFT-D minimizations of the two models represented as red and blue in Fig. 7 ▶ converge to identical minima, with the unit-cell orientations related to each other by 180° rotation around *b*, confirming the symmetry and energetic viability of this twinning mechanism.

Since the metric symmetry of DH-I is very close to monoclinic, the twinning is approximately merohedral (*i.e.* the Bragg peaks in the twin components are approximately overlapped). However, the γ angle deviates sufficiently from 90° for the twinning to be apparent as split peaks in the diffraction pattern, as described. Attempts at two-component integration of the single-crystal data were not successful, apparently due to the close overlap of the diffraction peaks. Post-analysis of the data integrated as a single component using *TWINROTMAT* in *PLATON* (Spek, 2009 ▶) was able to identify the twinning, and two-component refinement using the generated HKLF-5 format in *SHELXL* (Sheldrick, 2008 ▶) gave a refined batch scale factor of *ca*. 10%.

The applied preparation route for DH-II, by direct hydration of solid AH, prohibits the formation of diffraction-quality single crystals, and the DH-II structure was therefore considered using a combined PXRD/modelling approach. The ^13^C CP/MAS NMR spectra (Fig. 5 ▶) show that the arrangement of the naproxen molecules in DH-II must be very similar to AH, and the PXRD data for DH-II could be indexed on the basis of a unit cell that closely resembled AH (Table 1 ▶) to provide a very good Pawley fit. The *bc* plane of this unit cell is related to DH-I by doubling of the *c* axis, which is clearly required in order to accommodate the edge-on arrangement of naproxen molecules seen in AH. The first step to model the DH-II structure was therefore to create a supercell from the DH-I structure by doubling of the *c* axis (Fig. 9 ▶ *a*). The resulting enlarged unit cell could be transformed to the AH-type cell obtained from Pawley fitting by application of the matrix [1 0 −½ / 0 1 0 / 0 0 1]. The result corresponds to a supercell of the DH-I structure described in the AH-type cell setting (Fig. 9 ▶ *a*).

**Figure 8:**
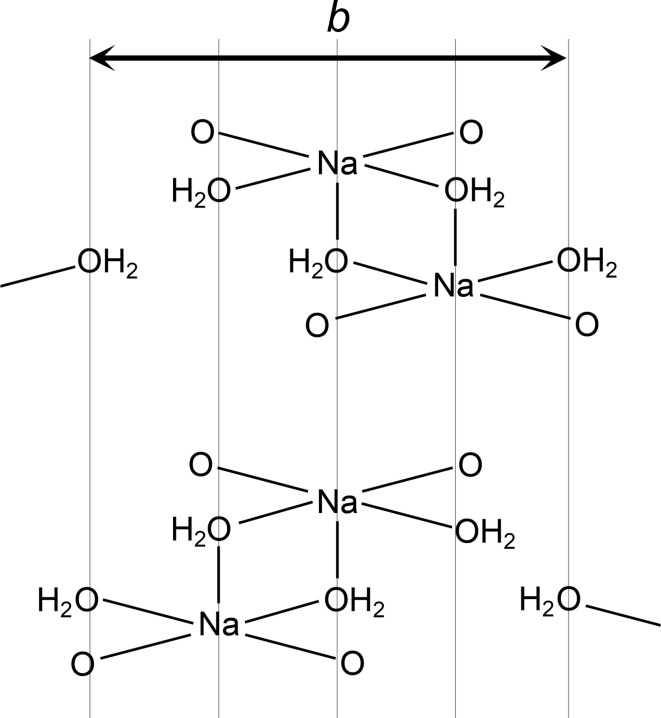
Schematic illustration of the alternative orientations for the Na^+^–(μ-OH_2_)_2_–Na^+^ units in a Na^+^/H_2_O section in DH-I. The positions of the water molecules do not change.

**Figure 9:**
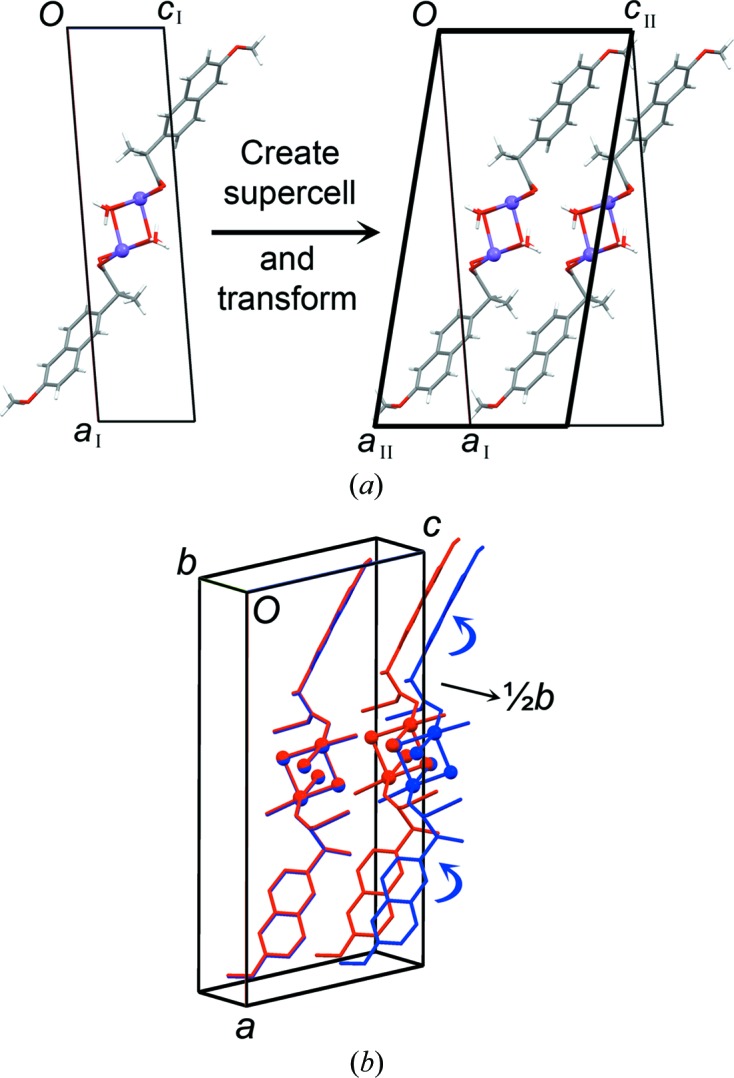
Modelling of the DH-II structure. (*a*) The DH-I unit cell is doubled and transformed (without changing the DH-I structure) into a setting comparable to that for AH. (*b*) One polymeric ribbon is shifted by ½*b* (red = before shift; blue = after shift), and the naphthalene rings are rotated to emulate the orientation in AH.

Comparison with the AH structure showed that the naproxen molecule positions in one of the polymeric ribbons closely reproduced those in AH, but the second polymeric ribbon should be shifted by ½*b* in order for the naproxen molecules to adopt positions similar to those in AH (Fig. 9 ▶ *b*). Crucially, this shift is permitted by the fact that the water molecules along the coordination polymers are distributed at intervals of approximately ½*b*, as shown in Fig. 8 ▶. Thus, the shift can be applied without changing the positions of the water molecules and without disrupting the hydrogen-bond network. Compelling evidence for the plausibility of the resulting structure is that the Na^+^/carboxylate/H_2_O region overlays essentially exactly the comparable region in the dihydrate of the sodium salt of ibuprofen (Ibu-DH; Zhang & Grant, 2005 ▶). The 3Q-MAS NMR spectra of DH-II and Ibu-DH also exhibit partially overlapping contours, induced by similar isotropic chemical shifts and EFG tensors (see supporting information). As a final step to model the DH-II structure, the naphthalene rings of both naproxen molecules in the shifted polymeric ribbon were rotated around the C5—C11 bond to emulate the ring positions in AH. The final model comprises Na^+^/carboxylate/H_2_O regions similar to those in Ibu-DH, with naproxen regions similar to those in AH. The broader lines in the ^13^C CP/MAS NMR spectra for DH-II and lack of singularities in the ^23^Na MAS NMR lineshapes (Fig. 5 ▶) indicate a higher degree of disorder compared with both Ibu-DH and AH.

**Figure 10:**
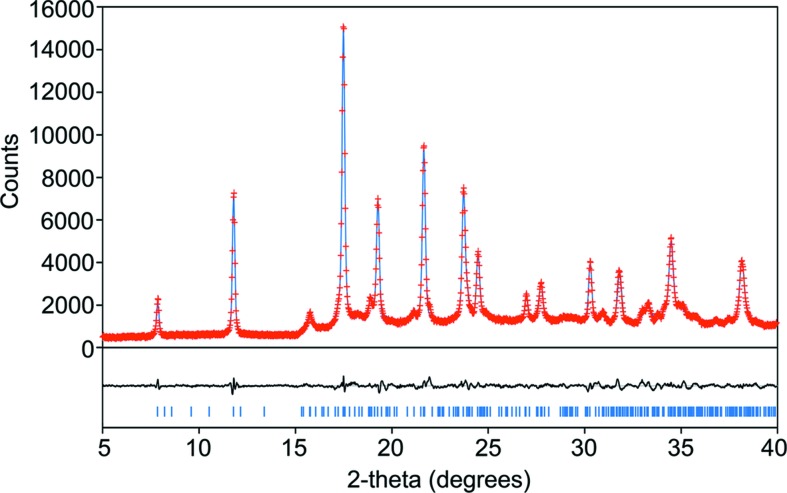
Rietveld refinement for DH-II against laboratory PXRD data (red crosses = measured points, blue line = calculated, black line = difference, blue ticks = Bragg peak positions). The intense low-angle (100) peak at 2θ = 3.94° was partially obscured, so is omitted from the refinement.

The DH-II model was subjected to DFT-D minimization in space group *P*1, initially with the unit-cell parameters constrained to those from the Pawley fit, then with the unit-cell parameters free to optimize. The minimized structure remained practically unchanged compared with the starting model, verifying that it is a viable energetic minimum. Finally, the minimized structure was used as the starting point for Rietveld refinement against the PXRD data, producing the fit illustrated in Fig. 10 ▶. The combination of the DFT-D minimization, satisfactory Rietveld fit and close similarity of the ^13^C NMR for DH-II and AH provide strong evidence for the validity of the proposed DH-II structure. The observed ‘modularity’ of the naproxen sections in AH and the Na^+^/carboxylate/H_2_O regions in sodium ibuprofen dihydrate provides a further intuitive indication that the structure is correct.

The DH-II structure also exhibits extensive pseudosymmetry. The naproxen molecules conform approximately to space group *P*2_1_, as for AH, although DH-II contains both molecular conformations shown in Fig. 3 ▶. The Na^+^/carboxylate/H_2_O regions alone conform very closely to the space group 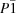 (as for racemic Ibu-DH; Zhang & Grant, 2005 ▶), with a unit-cell volume half that of the DH-II cell (see supporting information). The line broadening seen in the ^13^C NMR, and the multiple resonances for C11 and C13, can be attributed to the presence of the two alternative molecular conformations (Fig. 3 ▶). The resonances for C13 appear to be a superposition of the two resonances seen for AH and the single resonance for MH (or DH-I), as would be expected. The shoulder on the low-field side of the C14 resonance seems to be due to C11. The local environments of all C14 methyl groups are equivalent to those in AH, so variation in the environment of C14 does not account for this shoulder. Instead, the splitting seen for the C11 resonance in AH appears to be increased in DH-II so that the lower-field C11 resonance appears as the low-field shoulder on the C14 resonance.

### 3.5. Structural basis for the observed hydration/dehydration pathways   

The established structures for DH-I and DH-II provide an effective basis to rationalize the observed hydration/dehydration pathways in the sodium naproxen anhydrate–hydrate system. Both the AH↔DH-II and MH↔DH-I transformations can be referred to as topotactic, since they retain comparable crystallographic lattices. The topotactic transformations should have relatively low activation energies compared with the non-topotactic transformations, and they are observed to operate at room temperature or below. Thus, hydration of AH at 25°C proceeds directly to DH-II, and dehydration of DH-II under vacuum at −5°C proceeds directly to AH. Above room temperature, the non-topotactic pathways become viable as their higher activation barriers can be thermally overcome. Thus, AH transforms sequentially to MH then to DH-I at 50°C, while dehydration of DH-II at 40°C produces mainly MH. In this respect, it is notable that DH-II can be stored indefinitely at 25°C/55% relative humidity, where the non-topotactic pathways are not accessible. However, DH-II undergoes transformation to DH-I if it is stored at 50°C/80% relative humidity.

**Figure 11:**
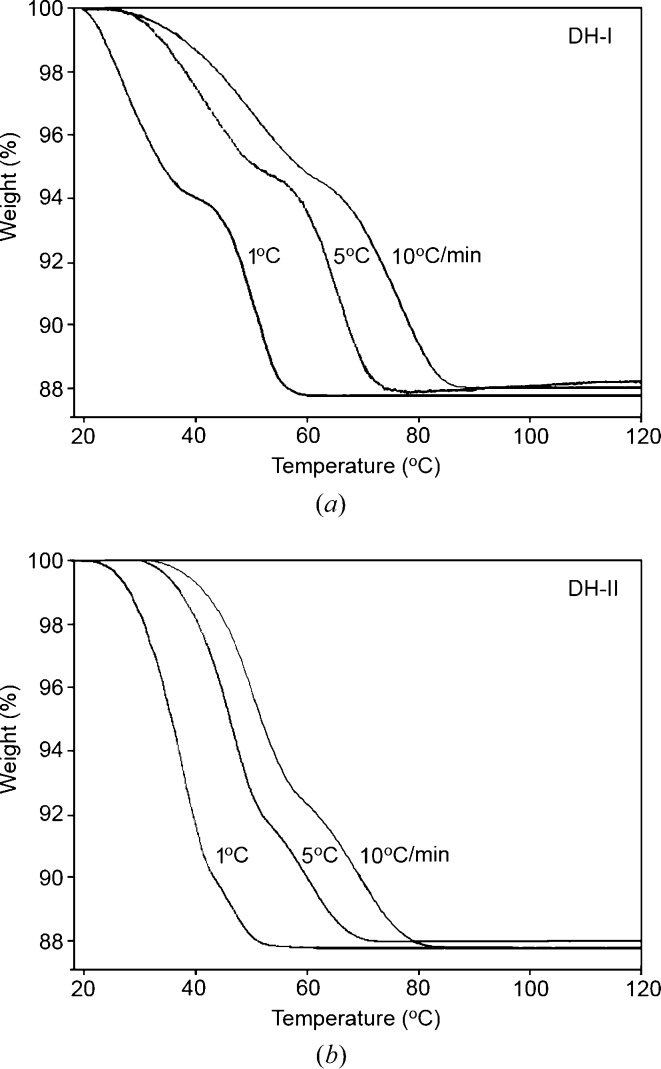
Thermogravimetric analysis (TGA) measured for (*a*) DH-I and (*b*) DH-II at heating rates 1, 5 and 10°C min^−1^.

In thermogravimetric analysis (TGA; Fig. 11 ▶), both DH-I and DH-II show an intermediate plateau corresponding to MH, but the shapes of the TGA curves are different, and they exhibit a different dependence on the heating rate (Fig. 11 ▶). For DH-I, the shape of the curve remains qualitatively similar at heating rates of 1, 5 or 10°C min^−1^, with the plateau occurring consistently around 93% weight, as expected for MH. For DH-II, the shape of the curve changes as a function of heating rate. As the heating rate is decreased, the plateau becomes less pronounced, and it moves progressively to lower weight %.

With the established structural information, we interpret the TGA data as follows:

(i) For DH-I, topotactic transformation to MH operates initially, while non-topotactic transformation to AH is activated only above a threshold temperature. In the TGA curve at 1°C min^−1^ the DH-I→MH transformation is essentially complete by 40°C, then the MH → AH transformation begins just below 50°C. Thus, the curve at 1°C min^−1^ approaches a step function. At higher heating rates, the plateau is less well defined because the non-topotactic MH → AH transformation is activated before the DH-I → MH transformation is complete (*i.e.* MH that is formed can transform immediately to AH).
(ii) For DH-II, topotactic transformation to AH operates initially, while the non-topotactic pathway *via* MH is activated only above a threshold temperature. At 1°C min^−1^, the DH-II → AH transformation is almost complete before the non-topotactic threshold temperature is reached, so the plateau for MH is small and it appears at a lower weight % because the MH produced from the DH-II that remains at that time comprises only a small fraction of the total sample. At higher heating rates, the non-topotactic pathway is activated while there is still a significant amount of DH-II present, so the plateau for MH appears more pronounced and closer to the expected 93 wt %.
(iii) Malaj *et al.* (2009 ▶) have previously reported two-step dehydration for DH-I and one-step dehydration for DH-II. They noted that the first step for DH-I has a comparable kinetic profile to the single step for DH-II. We interpret this as the kinetic profile for topotactic dehydration. The second-step dehydration of DH-I was fitted by Malaj *et al.* to a different empirical rate equation with the implication of a different physical mechanism. We interpret this as the non-topotactic dehydration of MH to AH.

For dehydration of DH-II to AH, it is interesting to consider the sequential DH-II → MH → AH pathway, which involves two non-topotactic steps and operates above room temperature. For the DH-II → MH step, we might speculate on whether the transformation occurs directly, or sequentially *via* DH-I. Conceptually, this depends on the rate for re­arrangement of the naproxen molecules from edge-on (in DH-II) to parallel (in DH-I), compared with the rate at which the water stoichiometry changes from dihydrate to monohydrate. If the naproxen rearrangement occurs before the water stoichiometry changes, the transformation proceeds *via* DH-I. If the stoichiometry changes simultaneously with the naproxen rearrangement, the transformation proceeds directly from DH-II to MH. The DFT-D minimized structures of MH and DH-I have essentially identical unit-cell volumes (see supporting information), which indicates that the packing arrangement is governed by the naproxen molecules and that the Na^+^/H_2_O region of either MH or DH-I can be accommodated within the same framework of naproxen molecules. The implication is that the hydrate stoichiometry for a crystalline material having the MH/DH-I structure could be continuously variable between MH and DH. In this case, the distinction between direct DH-II → MH or sequential DH-II → DH-I → MH transformation has little practical meaning, and the process is better represented as DH-II → [DH-I ↔ MH]. There is also a possibility that the water stoichiometry could change from DH to MH before any naproxen rearrangement occurs. This would produce a polymorph of MH having the edge-on naproxen structure (*i.e.* ‘MH-II’). We examined this possibility using PXRD and ss-NMR under various *in situ* dehydration conditions, but we did not find any evidence for such a phase. Likewise, we did not find any evidence for an AH phase having a parallel naproxen arrangement.

## 4. Conclusions   

The structural data provided here for the sodium naproxen anhydrate–hydrate system enable us to rationalize the complex transformation pathways that have previously been observed. The key is to establish the topotactic and non-topotactic nature of the various transformations. For this exercise to be effective, it is clearly important to have structural information for all involved solid phases. For DH-II in particular, the required solid-state preparation route hinders growth of suitable single crystals, and the combined modelling/PXRD/*S*-NMR approach becomes crucially important. Although the experimental and computational effort required to apply these techniques is significantly greater than that for a contemporary single-crystal X-ray analysis, the rewards for understanding systems of this type are significant.

## Supplementary Material

Crystal structure: contains datablock(s) DH1, DH2. DOI: 10.1107/S2052252514015450/ed5002sup1.cif

Structure factors: contains datablock(s) DH1. DOI: 10.1107/S2052252514015450/ed5002DH1sup3.hkl

Rietveld powder data: contains datablock(s) DH2. DOI: 10.1107/S2052252514015450/ed5002DH2sup4.rtv

CIF with DFT-D minimised structures for AH, MH, DH-I, DH-II. DOI: 10.1107/S2052252514015450/ed5002sup2.txt

Experimental details and additional figures for SS-NMR; reconstructed precession images for DH-I; displacement ellipsoid plot for DH-I; details of Rietveld refinements; comparison of DH-II and sodium ibuprofen dihydrate. DOI: 10.1107/S2052252514015450/ed5002sup5.pdf

CCDC references: 1019881, 1019882

## Figures and Tables

**Table 1:** Summary of the crystallographic information for AH, MH, DH-I and DH-II

	AH†	MH	DH-I	DH-II
Source	Kim *et al.* (2004 ▶)	Kim *et al.* (1990 ▶)	This work	This work
Formula	Na^+^[C_14_H_13_O_3_]^−^	Na^+^[C_14_H_13_O_3_]^−^·H_2_O	Na^+^[C_14_H_13_O_3_]^−^·2H_2_O	Na^+^[C_14_H_13_O_3_]^−^·2H_2_O
Formula weight	252.2	270.3	288.3	288.3
*T* (K)	298	291	150	298
Crystal system	Monoclinic	Monoclinic	Triclinic	Triclinic
Space group	*P*2_1_	*P*2_1_	*P*1	*P*1
*Z*/*Z*′	4/2	2/1	2/2	4/4
*a* (Å)	20.823 (6)	21.177 (6)	22.281 (9)	22.750 (6)
*b* (Å)	5.9346 (16)	5.785 (2)	5.811 (2)	5.747 (3)
*c* (Å)	9.969 (3)	5.443 (2)	5.435 (2)	10.866 (3)
α (°)	90	90	89.53 (2)	89.61 (4)
β (°)	102.025 (5)	91.41 (3)	85.53 (2)	98.20 (1)
γ (°)	90	90	92.61 (1)	92.11 (6)
*V* (Å^3^)	1204.9 (6)	666.6 (5)	700.8 (5)	1405.2 (8)
Density (g cm^−3^)	1.391	1.346	1.366	1.363

† Unit cell transformed compared with Kim *et al.* (2004 ▶).

**Table 2:** ^23^Na NMR isotropic chemical shifts (δ_iso_), quadrupolar coupling constants (*C*_Q_) and asymmetry parameters (*η*_Q_) for AH, MH, DH-I and DH-II

	δ_iso_ (p.p.m.)	*C*_Q_ (MHz)	η_Q_	Relative abundance
AH (site 1)†	2.7	3.17	0.57	0.55
AH (site 2)†	2.7	2.86	0.74	0.45
MH‡	4.1	1.04	0.54	–
DH-I‡	−0.8	2.95	0.25	–
DH-II‡ §	−1.7	2.30	0.49	0.5
	0.0	2.50	0.40	0.5
Ibuprofen-DH	0.0	2.80	0.20	–

† Fitted to spectra recorded at 9.4 and 16.4 T.

‡ Fitted to spectra recorded at 9.4 T.

§ An approximate fit is based on the two listed sites.
