# Supplementary material for: Structural basis for the transformation pathways of the sodium naproxen anhydrate–hydrate system
Source: IUCrJ. 2014 Aug 20;1(Pt 5):328–37. doi: 10.1107/S2052252514015450 (PMC4174875; doi:10.1107/S2052252514015450)
Supplement: Supplementary file 5 [file m-01-00328-sup5.pdf]

IUCrJ

Volume 1 (2014)

Supporting information for article:

Structural basis for the transformation pathways of the sodium naproxen anhydrate–hydrate system

Andrew D. Bond, Claus Cornett, Flemming H. Larsen, Haiyan Qu, Dhara Raijada and Jukka Rantanen

Structural basis for the transformation pathways of the sodium naproxen anhydrate-hydrate system

Andrew D. Bond, Claus Cornett, Flemming H. Larsen, Haiyan Qu, Dhara Raijada, and Jukka Rantanen

Corresponding author. E-mail: andrew.bond@sund.ku.dk

Supporting Information

S1. Solid-state NMR: experimental details

S2. Experimental and calculated ^{23}Na MAS NMR spectra

S3. ^{23}Na 3Q-MAS NMR spectra of sodium (*S*)-ibuprofen dihydrate and DH-II

S4. Reconstructed precession images for single-crystal data of DH-I

S5. Displacement ellipsoid plot for single-crystal structure of DH-I

S6. Rietveld refinement

S7. Relationship between DH-II and sodium (*S*)-ibuprofen dihydrate

References

S1. Solid-state NMR: experimental details

Solid-state NMR spectra were recorded on a Bruker Avance 400 spectrometer operating at Larmor frequencies of 100.62, 105.85 and 400.13 MHz for ^{13}C, ^{23}Na and ^{1}H, respectively, using a double-tuned CP/MAS probe equipped for 4 mm (o.d.) rotors.

^{13}C CP/MAS spectra were recorded using a contact time of 8.5 ms, a spin-rate of 12 kHz, a recycle delay of 8s, an acquisition time of 40.9 ms during which ^{1}H TPPM decoupling [Bennett, 1995] (80 kHz rf-field strength) was employed, 256 scans and a temperature of 313K. Cross polarization was carried out using variable amplitude CP [Peersen, 1993] with a maximum rf-field strength of 80 kHz for both ^{1}H and ^{13}C.^{13}C chemical shifts were referenced to an external sample of α-glycine (carbonyl group) at 176.5 ppm.

Single-pulse ^{23}Na MAS NMR spectra were recorded using a 1.8 μs pulse (69.4 kHz rf-field strength), spin-rate of 12 kHz, a recycle delay of 16 s, 32 scans, a temperature of 313 K, an acquisition time of 40.9 ms during which ^{1}H TPPM decoupling (80 kHz rf-field strength) were employed. ^{23}Na chemical shifts were referenced to an external sample of 1.0 M NaCl (aq) (0.0 ppm).

^{23}Na 3Q-MAS spectra were recorded using a z-filtered split-t1 3Q-MAS exp [Brown, 1997] employing a 3Q-excitation pulse of 18.7 μs, a 3Q-to-1Q conversion pulse of 37.1 μs (rf-field strength of 69.4 kHz for both) and two 5.0 μs (rf-field strength of 25 kHz) pulses for the z-filter, a recycle delay of 4 s, a spin-rate of 12 kHz, a temperature of 313 K and 192 or 768 scans for each of the 72 t1-increments of 83.33 μs in the isotropic dimension. Subsequently data were processed using the *NMRpipe* software [Delaglio, 1995].

One single-pulse ^{23}Na MAS spectrum of NSAH was recorded on a Bruker Avance-III 700 spectrometer operating at a Larmor frequency of 185.15 MHz for ^{23}Na using a triple-tuned probe equipped for 4 mm (o.d.) rotors. This spectrum was recorded using a pulse width of 1.0 μs (rf-field strength: 41.7 kHz), a recycle delay of 4 s, a spin-rate of 12 kHz, 1024 scans, and an acquisition time of 8.24 ms.

S2. Experimental and calculated ^{23}Na MAS NMR spectra

[Calculated spectra employ the parameters listed in Table 2 of the main manuscript]

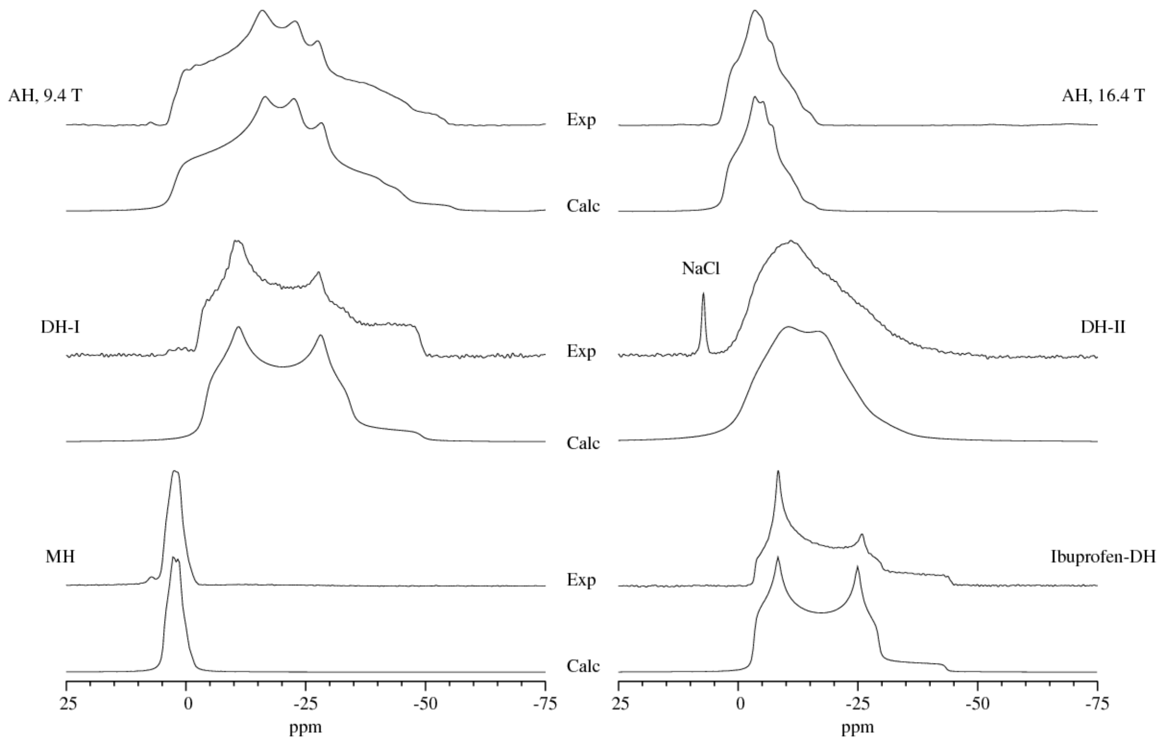

S3. ^{23}Na 3Q-MAS NMR spectra of sodium (*S*)-ibuprofen dihydrate (A) and DH-II (B)

[A horizontal line at the shift of Ibuprofen-DH in the isotropic dimension is inserted as a guide to the eye. The asterisk denotes an MH impurity in the DH-II sample]

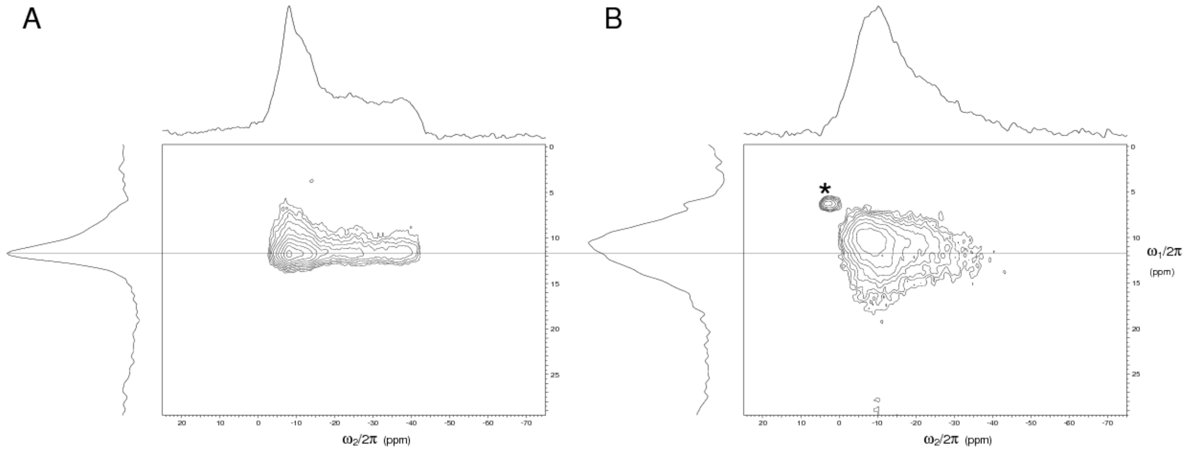

S4. Reconstructed precession images for single-crystal data of DH-I

[Peak predictions correspond to: *a* = 22.281, *b* = 5.811, *c* = 5.435 Å, α = 89.5, β = 85.5, γ = 92.6°]

(0*kl*)

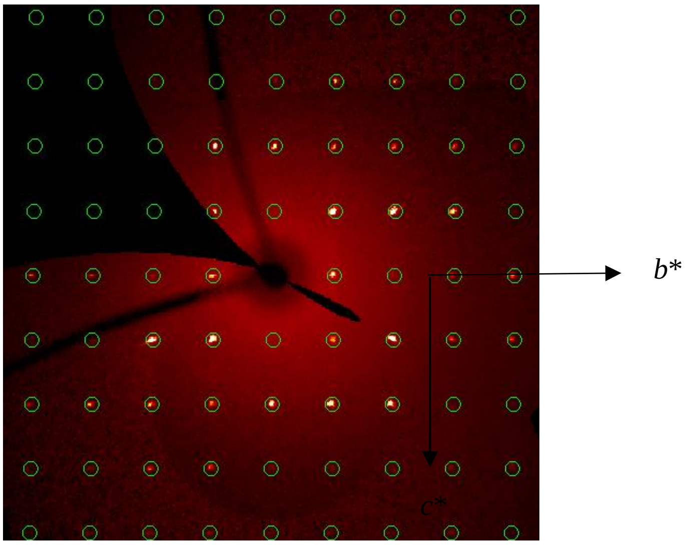

(1*kl*)

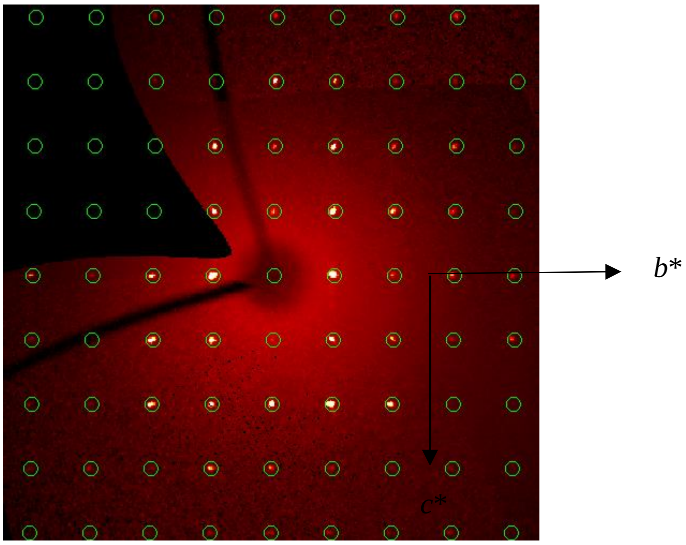

(2*kl*)

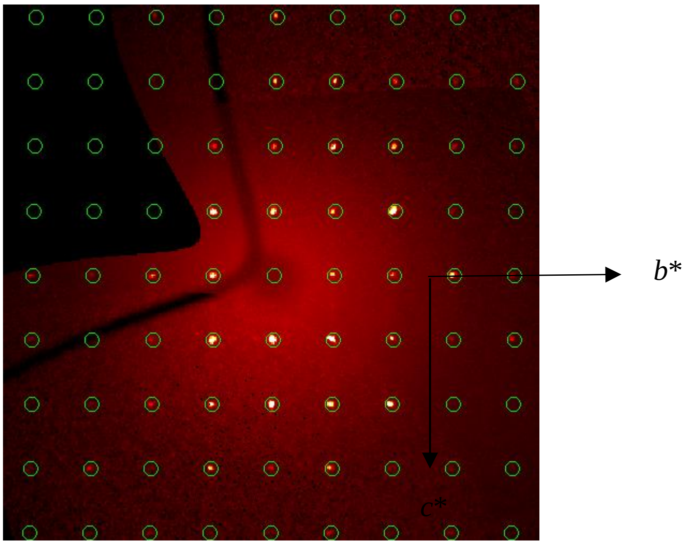

S4. Reconstructed precession images for single-crystal data of DH-I

[Peak predictions correspond to: a = 22.281, b = 5.811, c = 5.435 Å, α = 89.5, β = 85.5, γ = 92.6°]

(*h*0*l*)

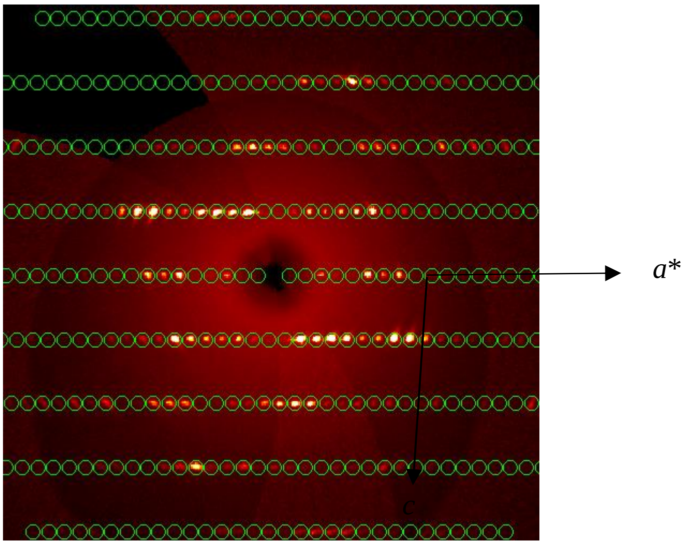

(*h*1*l*)

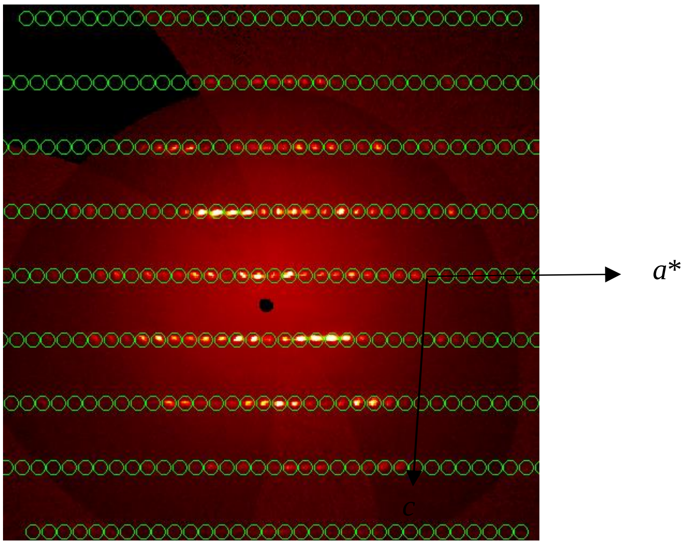

(*h*2*l*)

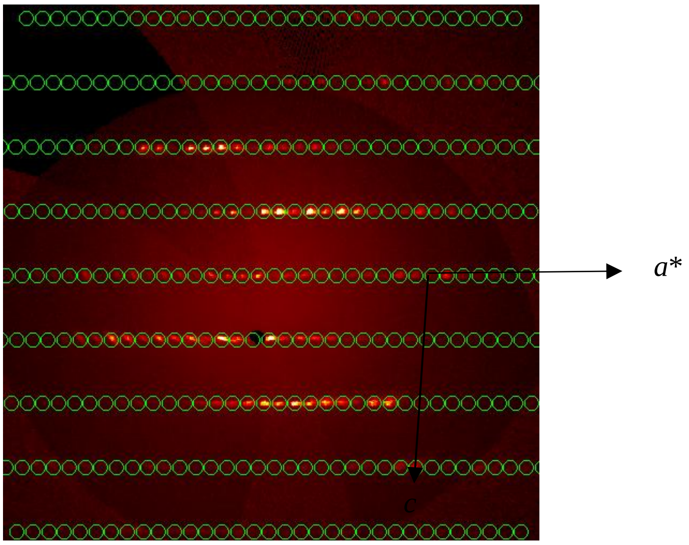

S4. Reconstructed precession images for single-crystal data of DH-I

[Peak predictions correspond to: *a* = 22.281, *b* = 5.811, *c* = 5.435 Å, α = 89.5, β = 85.5, γ = 92.6°]

(*hk*0)

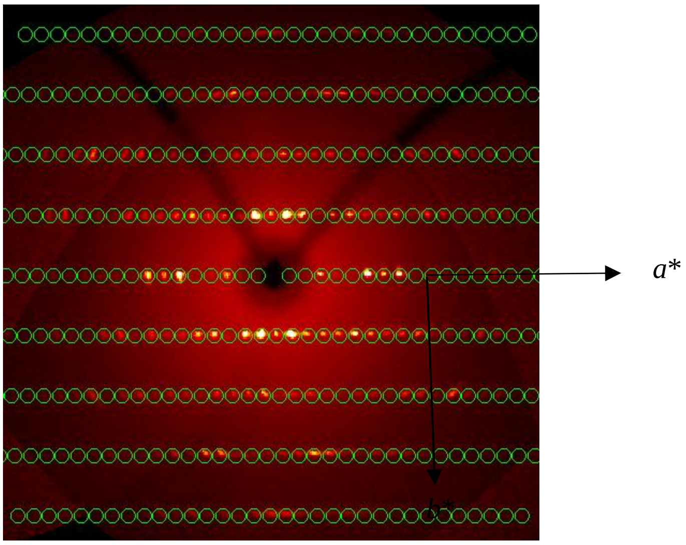

(*hk*1)

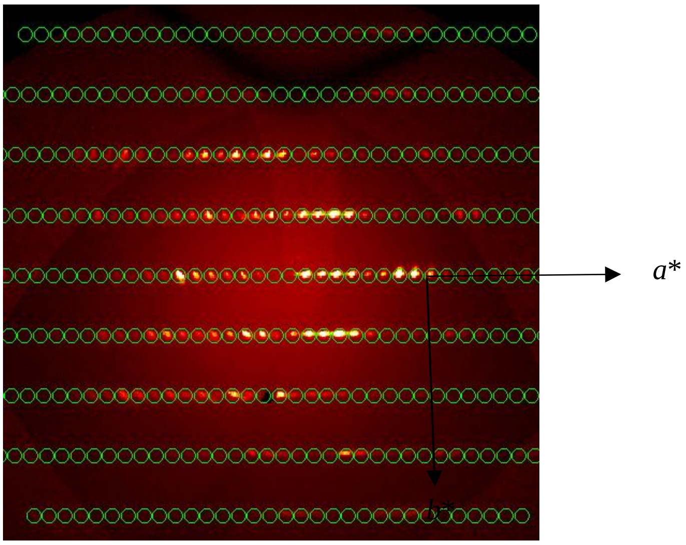

(*hk*2)

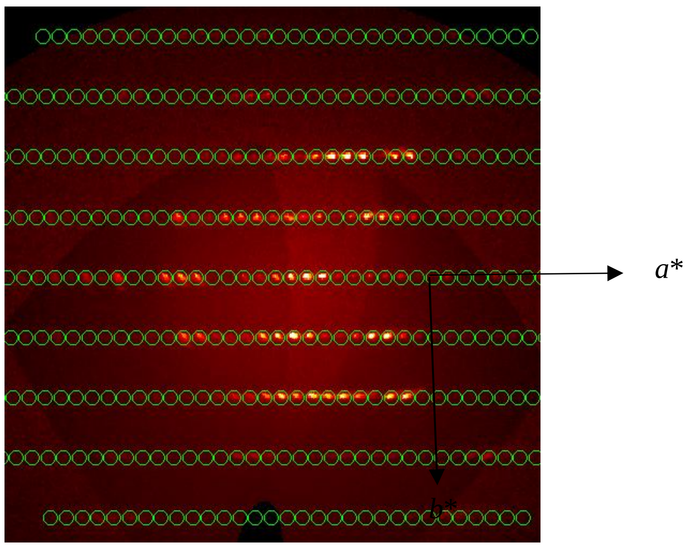

S5. Displacement ellipsoid plot for single-crystal structure of DH-I

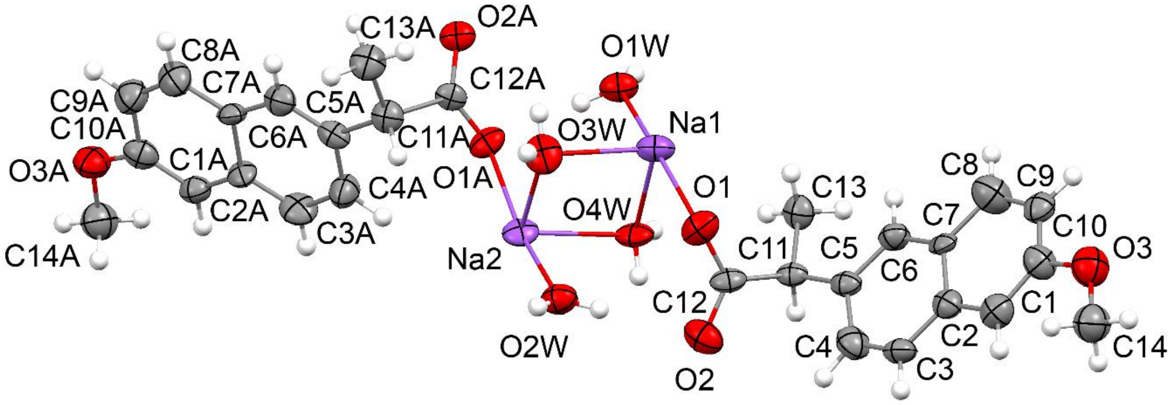

S6. Rietveld refinement

Rietveld refinements were carried out using *TOPAS* Academic ver. 4.1 (Coelho, 2007). The interface in *DASH* (David et al., 2006) was used to construct the initial input files. The starting point for each refinement was the DFT-D minimized structure. Tight restraints were generated for all bond distances and angles, and the naphthalene rings of the naproxen molecules were also tightly restrained to be planar. All coordinates (including H atoms) were refined with a common isotropic displacement parameter for non-H atoms, and with U(iso) for the H atoms equal to 1.2 times the refined parameter. The background was modelled with a 20-term Chebyshev polynomial and peak asymmetry was refined using spherical harmonics. A correction for preferred orientation was applied in each case using the March-Dollase model. The refinement strategy allows the fit to the experimental PXRD pattern to be optimized, while keeping the structure close to the DFT-D optimized structure. The result is a sensible model, close to that expected from a single-crystal refinement, with a measure of how well that model fits the experimental data.

S6.1. Rietveld refinement for AH

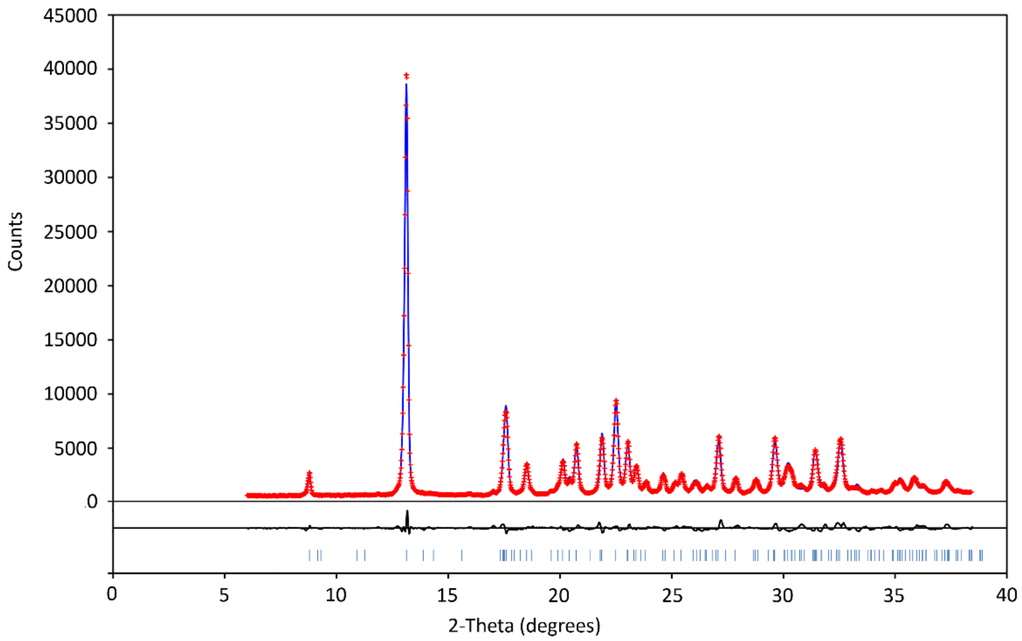

	Rp (%)	Rwp (%)	χ^2
Before background subtraction	4.922	6.600	2.98
After background subtraction	8.346	9.990	

S6.2. Rietveld refinement for MH

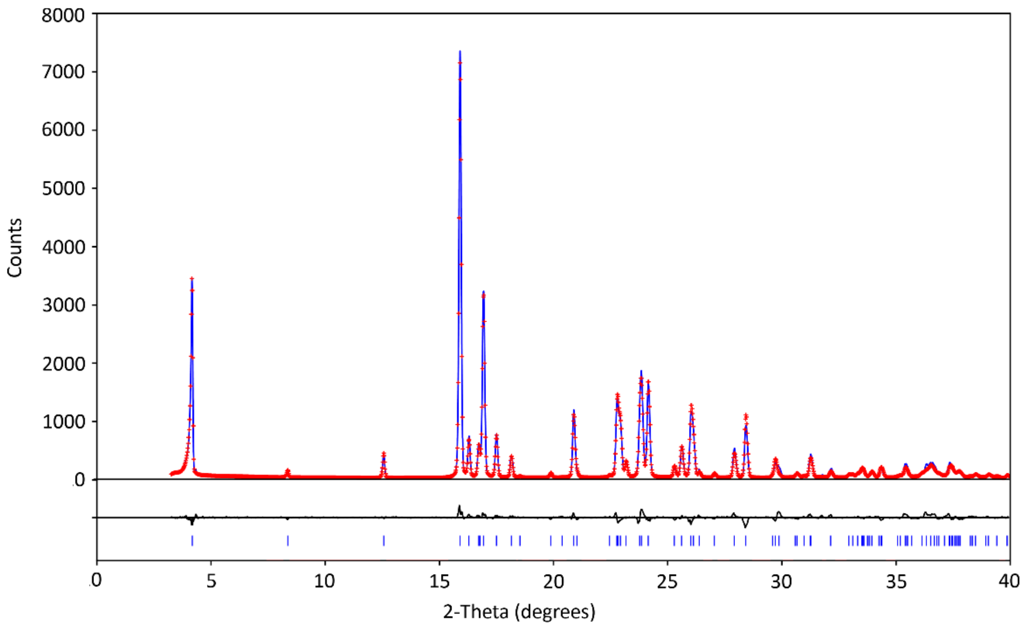

	Rp (%)	Rwp (%)	χ^2
Before background subtraction	5.021	7.496	1.03
After background subtraction	6.800	9.313	

S6.3. Rietveld refinement for DH-I

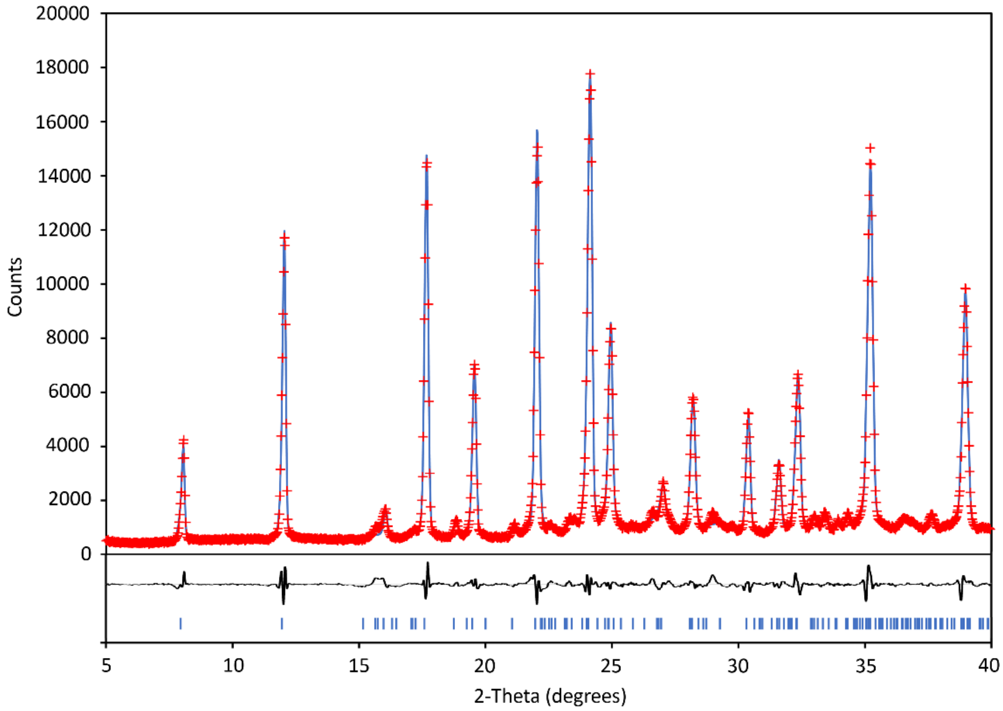

	Rp (%)	Rwp (%)	χ^2
Before background subtraction	4.774	6.800	2.99
After background subtraction	3.700	4.888	

S6.4. Rietveld refinement for DH-II [difference curve also shown in the main paper]

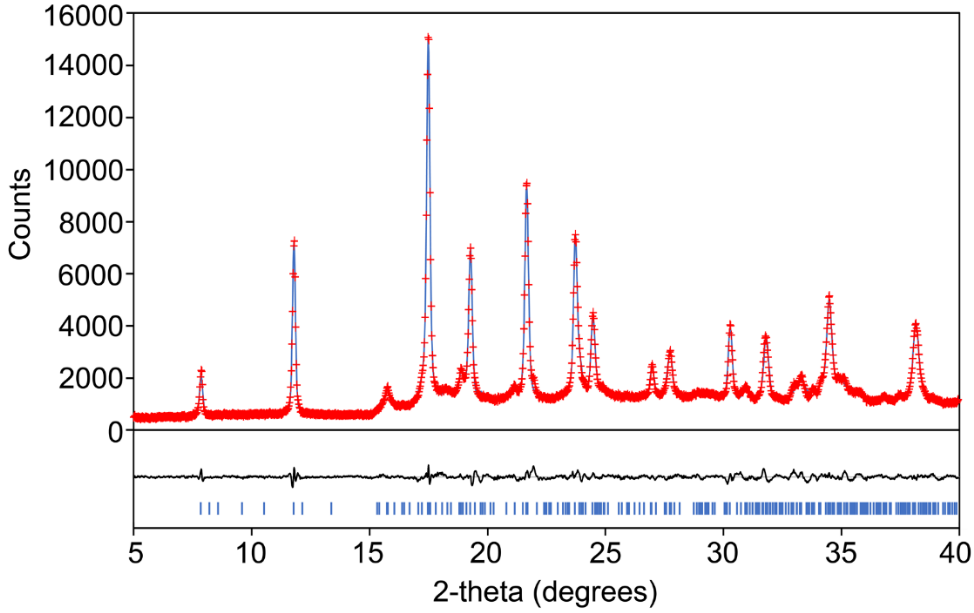

	Rp (%)	Rwp (%)	χ^2

Before background subtraction	3.916	5.267	2.582
After background subtraction	5.438	7.031	

S7. Relationship between DH-II and sodium (*S*)-ibuprofen dihydrate

[CSD: KATNOJ (enantiomeric ibuprofen) and KASVEG (racemic) are isostructural]

Overlay of DH-II (red) and KASVEG (green). Only Na, H_2O and carboxylate groups are shown. The right-hand diagram shows that the Na/H_2O/carboxylate region of DH-II conforms to space group *P*–1, as for KASVEG. [In the DH-II cell, space group is formally *A*–1]:

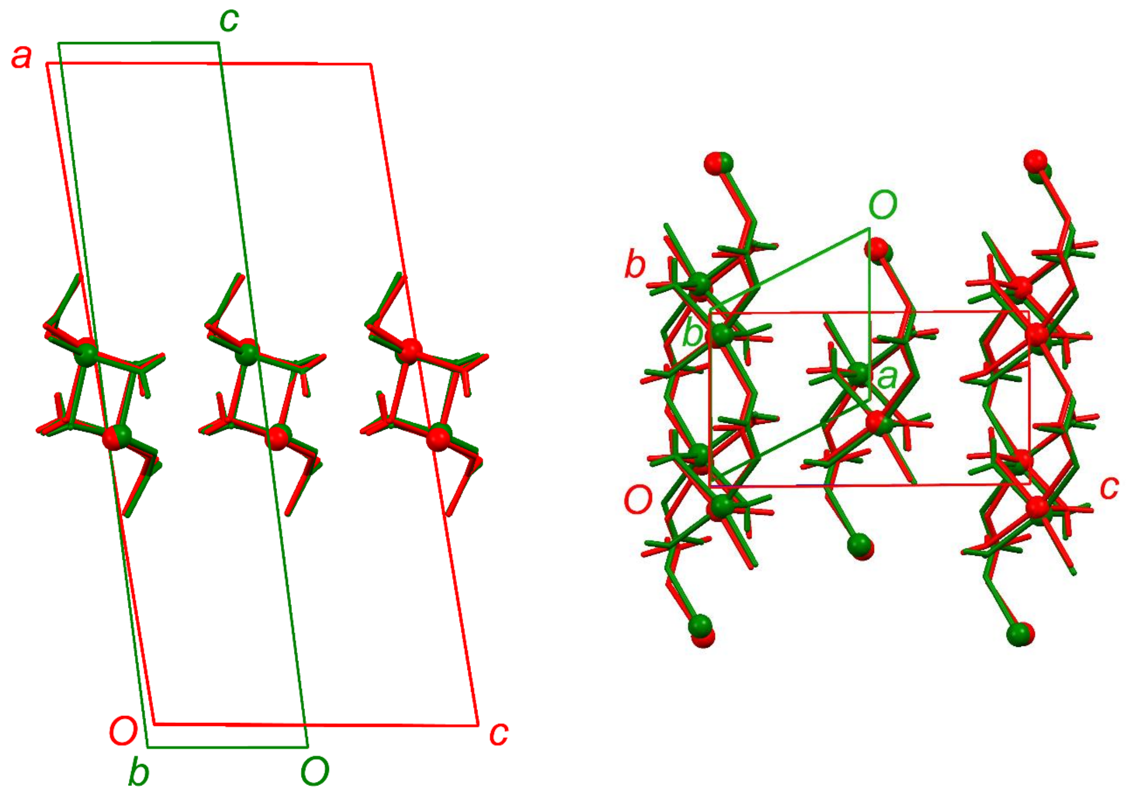

Relationship between the unit cells:

$$\begin{pmatrix} 0 & 0 & -1 \\ 1 & 0 & 0 \\ -1 & 2 & 0 \end{pmatrix}$$

KASVEG	5.740	6.028	23.830
	83.46	89.24	63.15

→

23.830	5.740	10.761
91.57	96.93	90.76

DH-II	22.750	5.747	10.866
	89.61	98.20	92.11

References

Delaglio, F.; Grzesiek, S.; Vuisiter, G. W.; Zhu, G.; Pfeifer, J.; Bax, A. *J. Biomol. NMR*, **1995**, *6*, 277.

Brown, S. P.; Wimperis, S. *J. Magn. Reson.* **1997**, *128*, 42.

Peersen, O. B.; Wu, X.; Kustanovich, I.; Smith, S. O. *J. Magn. Reson. A* **1993**, *104,* 334.

Bennett, A. E.; Rienstra, C. M.; Auger, M.; Lakshmi, K. V.; Griffin, R. G. *J. Chem. Phys.* **1995**, *103*, 6951.
